# Circulating and local nuclear expression of survivin and fibulin-3 genes in discriminating benign from malignant respiratory diseases: correlation analysis

**DOI:** 10.1042/BSR20203097

**Published:** 2021-01-06

**Authors:** Mohammed H. Hassan, Sawsan Abuhamdah, Mohamed Abdel-Bary, Mohammed Wahman, Tarek Hamdy Abd-Elhamid, Morris Beshay, Karam Mosallam, Bakheet E.M. Elsadek

**Affiliations:** 1 Department of Medical Biochemistry, Faculty of Medicine, South Valley University, Qena, Egypt; 2 College of Pharmacy, Al Ain University, Abu Dhabi, UAE; 3 Department of Biopharmaceutics and Clinical Pharmacy, Faculty of Pharmacy, The University of Jordan, Amman, Jordan; 4 Department of Cardio-Thoracic Surgery, Faculty of Medicine, South Valley University, Qena, Egypt; 5 Department of Oncology and Nuclear Medicine, Faculty of Medicine, South Valley University, Qena, Egypt; 6 Department of Histology and Cell Biology, Faculty of Medicine, Assiut University, Assiut, Egypt; 7 Department of General Thoracic Surgery, Protestant Hospital of Bethel Foundation, Burgsteig 13, Bielefeld, Germany; 8 Department of Biochemistry, Faculty of Pharmacy, Al-Azhar University, Assiut Branch, 71524 Assiut, Egypt

**Keywords:** Fibulin-3, lung cancer, malignant pleural mesothelioma, Survivin

## Abstract

Survivin is an inhibitor of apoptosis as well as a promoter of cell proliferation. Fibulin-3 is a matrix glycoprotein that displays potential for tumor suppression or propagation. The present study aimed to validate the expression levels of survivin and fibulin-3 in benign and malignant respiratory diseases. This case–control study included 219 patients categorized into five groups. Group A included 63 patients with lung cancer, group B included 63 patients with various benign lung diseases, group D included 45 patients with malignant pleural mesothelioma (MPM), and group E included 48 patients with various benign pleural diseases. Group C included 60 healthy individuals (control group). Serum survivin and fibulin-3 levels were measured by ELISA, whereas their nuclear expressions in the lung and pleura were assessed via Western blot analysis. The results showed significantly higher survivin serum levels and significantly lower fibulin-3 levels in group A compared with in group B and controls (*P*<0.001). There were significantly higher serum levels of survivin and fibulin-3 in group D compared with in group E and controls (*P*<0.001), consistent with observed nuclear survivin and fibulin-3 expression levels. Fibulin-3 was determined to have higher value than survivin in discriminating lung cancer from MPM (*P*<0.05). Survivin and fibulin-3 could be useful diagnostic markers for lung and pleural cancers, and fibulin-3 expression was particularly useful in differentiating lung cancer from MPM**.**

## Introduction

It is widely understood that pathological inhibition of apoptosis plays a significant role in the growth, progression, and resistance to therapy of cancer [1]. Survivin is a member of the inhibitor of apoptosis protein (IAP) family and comprises 142 amino acids with a molecular weight of 16.5 kDa. It is encoded by a single-copy gene located on human chromosome 17q25, with a dual role in cell proliferation and apoptosis prevention [2–4]. Survivin is expressed in the nucleus and/or cytoplasm of various malignant tumor cells. In the cytoplasm, survivin exerts an anti-apoptotic effect, but regulates cell proliferation in the nucleus [5,6].

Increased expression of fibulin-3 was found to inhibit TGF-β-induced epithelial–mesenchymal transition (EMT) and endothelial permeability in breast cancer, as well as cell morphology, proliferation, encroachment, adhesion, and migration [7,8], whereas the loss of expression or function of fibulin-3 promoted these TGF-β-mediated effects [9]. Fibulin-3 up-regulation suppressed the invasion and migration of lung adenocarcinoma cells, as well asthe expression of EMT activators N-cadherin and Snail [10]. Fibulin-3 also suppresses extracellular signal-regulated kinase signaling, which results in the inhibition of Wnt*/*β-catenin signaling [11], as well as the inhibition of the upstream modifiers of glycogen synthase kinase 3β (GSK3β), such as insulin-like growth factor-1 receptor (IGF1R) and phosphoinositide 3-kinase (PI3K) [12]. Thus, fibulin-3appears to possess the capacity to act as either a tumor suppressor or an oncogene depending on the cellular context [13].

Studies addressing the diagnostic efficacies of both survivin and fibulin-3 in respiratory malignancies have reported conflicting results. The present study was conducted to analyze the local and circulating expression levels of these biomarkers in various benign and malignant lung and pleural diseases and to validate their utility in diagnosing and discriminating malignant lesions from benign lesions in the respiratory tract.

## Materials and methods

### Study design and participants

The current case–control study included 219 patients of both sexes with recently diagnosed benign and malignant respiratory diseases recruited from the Cardio-Thoracic Surgery and Oncology Departments of Qena University Hospitals, South Valley University, Egypt. Patients were categorized into four groups. Group A included 63 patients diagnosed with lung cancer, group B included 63 patients diagnosed with various benign lung diseases, group D included 45 patients diagnosed with malignant pleural mesothelioma (MPM), and group E included 48 patients diagnosed with various benign pleural diseases. In addition, 60 age- and sex-matched unrelated apparently healthy subjects served as a control group (group C). Patients with renal failure, hepatic failure, severely compromised cardiopulmonary systems, coagulopathy, or hemodynamic instability were excluded. The duration of the study was two years, from January 1, 2017 to December 30, 2019.

### Data collection

Full medical history and thorough clinical examinations were performed for every patient. Additionally, radiological assessments, including plain chest X-rays, CT scans, and routine laboratory investigations [serum lactate dehydrogenase (LDH), total protein, albumin, liver and kidney functions, erythrocyte sedimentation rate (ESR), and complete blood count (CBC)] were performed. Multiple lung biopsies were taken and sent for histopathological examination where indicated. Thoracentesis was performed in cases with demonstrated pleural effusion. Thoracoscopy was performed in patients with ambiguous cytology or pleural fluid analysis, and multiple pleural biopsies were subsequently taken for histopathological examination. Malignant pleural effusion was diagnosed if pleural fluid cytology or pleural biopsy findings were positive for malignancy [14]. Staging of MPM was performed using van Meerbeeck et al. [15], while that of lung cancer was assessed according to Lim et al. [16].

### Biochemical and molecular assays of survivin and fibulin-3

#### Blood samples

Five milliliters of venous blood was collected from each patient into serum separator gel tubes, and was allowed to clot at room temperature for 30 min before centrifugation for 15 min at 1000 × ***g***. The separated sera were stored in aliquots in 1 ml cryotubes at −80°C for future biochemical assays of survivin and fibulin-3.

#### ELISA assays of survivin and fibulin-3

Quantitative determination of serum survivin and fibulin-3 was achieved using commercially available ELISA assay kits supplied by Chongqing Biospes Co., Ltd, China (catalog numbers BYE3519 and BYEK2017, respectively). Assays were performed using a microplate ELISA reader (EMR 500, U.S.A.) according to the manufacturer’s protocol.

#### Western blotting analysis of local survivin and fibulin-3 expression levels

Lung and pleural biopsies were homogenized in ice-cold RIPA lysis buffer (Sigma-Aldrich, Milan, Italy) containing 1% protease inhibitor cocktail (Cell Signaling Technology, Inc., MA, U.S.A.) using a Potter-Elvehjem rotor-stator homogenizer (glass/Teflon homogenizer) and were preserved at −70°C for subsequent analysis of tissue nuclear survivin and fibulin-3 expression by Western blotting.

Proteins in each corresponding lung or pleural tissue homogenate were denatured at 95°C for 5 min in 2× Laemmli buffer, followed by the addition of 5% 2-mercaptoethanol. SDS–PAGE electrophoresis was performed by passing 50 µg protein per lane at 75 volts through a resolving gel (18% for surviving and 15% for fibulin-3), followed by 125 volts for approximately 2 h, and subsequent transfer to a PVDF membrane using a T-77 ECL semidry transfer unit (Amersham BioSciences U.K. Ltd) for 2 h. Immunoblotting was performed by incubating the PVDF membrane in TBS buffer containing 0.1% Tween (TBST) and 5% non-fat milk for 1 h at 4°C, followed by overnight incubation at 4°C with 1:1500 dilutions of rabbit anti-survivin polyclonal antibodies (Bioss Inc., Massachusetts, USA) and rabbit anti-fibulin-3 polyclonal antibodies(Novus Biologicals, LLC, Littleton, CO, U.S.A.). After washing with TBST buffer three times, each membrane was incubated at room temperature for 1 h with an alkaline phosphatase-conjugated goat anti-mouse secondary antibody (Novus Biologicals, LLC, Littleton, CO, U.S.A.) at a 1:5000 dilution, and then washed four times in TBST. The membrane-bound antibody was detected using a commercially available BCIP/NBT substrate detection Kit (Genemed Biotechnologies, Inc., CA, U.S.A.). Equivalent protein loading was confirmed for each lane by stripping and reblotting each membrane at 4°C against the mouse monoclonal anti-β-actin antibody (Santa Cruz Biotechnology, Inc., CA, U.S.A.) at a 1:5000 dilution. These analyses were replicated three times to ensure that the findings were reproducible. Quantification was performed using ImageJ software, and expressed as the band density relative to that of β-actin [17,18].

### Statistical analysis

Data entry and data analysis were performed using SPSS version 19 (Statistical Package for Social Science). Parametric data are presented as numbers, percentages, means, and standard deviations. The Chi-square test and Fisher’s exact test were used to compare qualitative variables. Independent *t*-tests were used to compare quantitative variables between two groups. ANOVA (*f*) test was used for comparison between three or more groups having quantitative variables that normally distributed. Pearson correlations were performed to measure the correlation between quantitative variables. The Medcalc Program was used to calculate sensitivity, specificity, and positive and negative predictive values via the area under the curve (AUC) (95% CI). *P* values were considered statistically significant when less than 0.05.

## Results

### Demographic data of the study groups

The current study was conducted in 219 age- and sex-matched patients (141 males and 78 females) with various benign and malignant lung and pleural diseases. These patients were categorized into four groups: group A included 63 patients diagnosed with lung cancer (42 males and 21 females, with a mean age of 57.67 ± 7.51); group B included 63 patients diagnosed with various benign lung diseases (39 males and 24 females, with a mean age of 51.62 ± 11.14); group D included 45 patients diagnosed with MPM (27 males and 18 females, with a mean age of 53.73 ± 4.95); and group E included48 patients diagnosed with various benign pleural diseases (33 males and 15 females, with a mean age of 52.13 ± 4.11). Sixty healthy controls (30 males and 30 females) age- and sex-matched with the study patients (mean age 55.5 ± 7.88 years old) comprised the control group C.

### Clinical data of the patient groups

Analysis of the clinical data of each patient in the groups diagnosed with benign respiratory diseases (groups B and E) revealed chronic lung abscesses and bronchiectasis. Postcavitary T.B lesions in 11patients (17.4%), emphysematous bulla in 25 cases (39.7%), and arteriovenous malformation in 5(8%) were discovered in group B. Among patients in group E, empyema was present in 15 (31.3%), chronic pleurisy was diagnosed in 24 (50%), and pleural fibroma was diagnosed in 9(18.8%).

Analysis of the tumor stage of patients diagnosed with malignant respiratory diseases (groups A and D) revealed that 27patients (42.9%) had stage-II, 6 (9.5%) had stage-III, and 30 (47.6%) had stage-IV lung cancer. Among those diagnosed with MPM, 12 patients (26.7%) had stage-III and 33 (73.3%) had stage-IV MPM. Adenocarcinoma was present in 36 cases (57.1%) and squamous cell carcinoma was present in 27 patients (42.9%) of patients diagnosed with non-small-cell lung cancer (NSCLC). Among the patients diagnosed with MPM, epithelial mesthothelioma in 30 cases (66.7%) and sarcomatoid mesthothelioma was present in 15 cases (33.3%).

### Survivin and fibulin-3 expression levels in the study groups

There were significantly higher serum levels of survivn among patients with lung cancer (681.93 ± 274.79 pg/ml) when compared with patients with benign lung diseases and controls (423.34 ± 239.73 and 269.22 ± 8.01 pg/ml, respectively; *P*<0.001), and significantly higher serum survivin levels among patients with benign lung diseases compared with among healthy controls (*P*=0.006). In contrast, there were significantly lower serum fibulin-3 levels among patients with lung cancer (3.56 ± 2.18 pg/ml) compared with among patients with benign lung diseases and controls (10.16 ± 7.83 and 8.99 ± 2.2 pg/ml, respectively) (*P*<0.001), with a non-significant difference in the serum levels of fibulin-3 between patients with benign lung diseases and controls (*P*=0.364) (Table 1).

**Table 1:** Mean ± Standard Deviation (SD) of serum survivin and fibulin-3 levels among patients with benign and malignant lung diseases compared with the control group

Measured biochemical markers	Group A (*N*=63)	Group B (*N*=63)	Group C (*N*=60)	*P1	*P2	*P3
**Serum survivin (pg/ml), (means ± SD)**	681.93 ± 274.79	423.34 ± 239.73	269.22 ± 8.01	<0.001**	0.006**	<0.001**
**Serum fibulin-3 (pg/ml), (means ± SD)**	3.56 ± 2.18	10.16 ± 7.83	8.99 ± 2.2	<0.001**	0.364	<0.001**

*P1 = A vs. C

*P2 = B vs C

*P3 = A vs B

**P<0.05, significant; *P>*0.05, non-significant.

**N.B:** Group A: patients with lung cancer; group B: patients with benign lung diseases; group C: control group.

There were significantly higher serum levels of survivin among patients with MPM (582.75 ± 71.91 pg/ml) when compared with among patients with benign pleural diseases and controls (259.7 ± 17.78 and 269.22 ± 8.01 pg/ml, respectively) (*P*<0.001), with a non-significant difference in the serum levels of survivin between patients with benign pleural diseases and controls (*P*=0.873). In addition, there were significantly higher serum fibulin-3 levels among patients with MPM (14.5 ± 3.07 pg/ml) when compared with among patients with benign pleural diseases and controls (8.9 ± 2.3 and 8.99 ± 2.2 pg/ml, respectively) (*P*<0.001), with a non-significant difference in the serum levels of fibulin-3 between patients with benign pleural diseases and controls (*P*=0.949) (Table 2).

**Table 2:** Mean ± Standard Deviation (SD) of plasma survivin and fibulin-3 levels among patients with benign and malignant pleural diseases compared with the control group

Measured biochemical markers	Group D (*N*=45)	Group E (*N*=48)	Group C (*N*=60)	*P1	*P2	*P3
**Serum survivin (pg/ml), (means ± SD)**	582.75 ± 71.91	259.7 ± 17.78	269.22 ± 8.01	<0.001**	0.873	<0.001**
**Serum fibulin-3 (pg/ml), (means ± SD)**	14.5 ± 3.07	8.9 ± 2.3	8.99 ± 2.2	<0.001**	0.949	<0.001**

*P1 = D vs. C

*P2 = E vs C

*P3 = D vs E

**P<0.05,significant; *P>*0.05, non-significant.

**N.B:** Group D: patients with malignant pleural mesothelioma; group E: patients with benign pleural disease; group C: control group.

**Figure 1:**
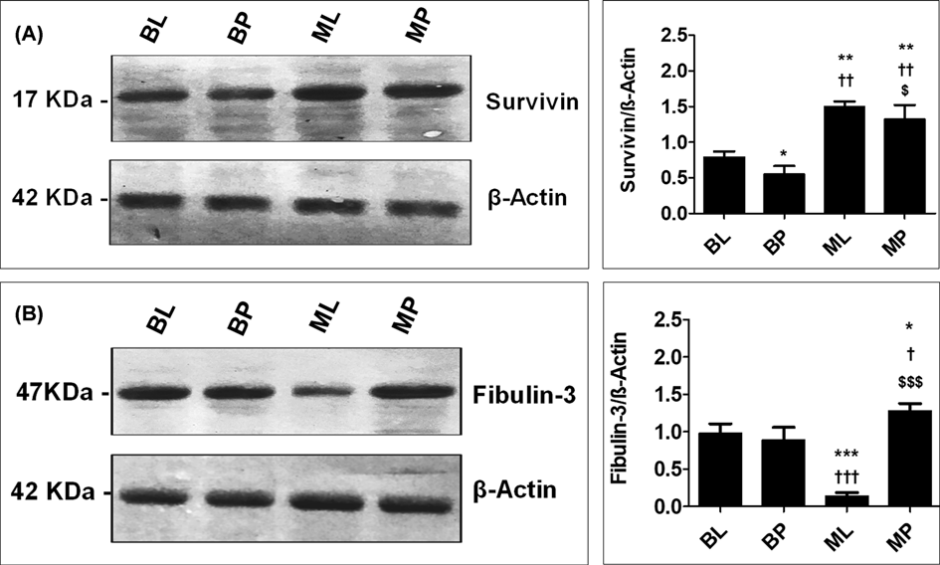
Representative Western blot analysis of survivin (A) and fibulin (B) expression in different groups β-Actin was used in parallel as internal control. The right panels represent the corresponding quantification of each analysis measured by the Image J software and expressed as the relative band density to β-actin. The levels of significance were accepted with *P*<0.05 and all relevant results were graphically displayed as mean ± SD (*n*=3). *, † and $ indicate significant changes from the BL, BP, and ML groups respectively. *, † and $ indicate significant change at *P*<0.05; **, †† and $$ indicate significant changes at *P*<0.01; ***, ††† and $$$ indicate significant changes at *P*<0.001. BL, benign lung diseases; BP, benign pleural diseases; ML, malignant lung diseases; MP, malignant pleural diseases.

Western blot analysis showed that local survivin and fibulin-3 expression levels in patients with benign and malignant respiratory diseases were consistent with the circulating levels. Local survivin levels were significantly higher among patients with lung cancer and MPM. Interestingly, local fibulin-3 levels among patients with lung cancer were significantly lower than among patients with MPM (Figure 1; Supplementary Figures S1 and S2).

Regarding to the mean serum survivin and fibulin-3 levels in the included patients with lung cancer or MPM in terms of tumor stage, the mean serum survivn and fibulin-3 (pg/ml) levels didn’t significantly differ among lung cancer patients with stage II (636.4 ± 161.0 and 3.146 ± 1.68, respectively) or III (424.7 ± 219.0 and 1.785 ± 0.68, respectively) or IV (774.3 ± 336.4 and 4.291 ± 2.54, respectively) with *P*>0.05 for all. Also, the mean serum survivn and fibulin-3 (pg/ml) levels didn’t significantly differ among MPM patients with stage III (626.5 ± 94.93 and 13.23 ± 4.11, respectively) or IV (568.8 ± 79.96 and 15.44 ± 1.95, respectively) with *P>*0.05 for all.

Additionally, there were no significant differences in the serum survivin and fibulin-3 levels in the included patients with lung cancer or MPM with respect to histological types, *P>*0.05 for all.

### Correlation between of survivin and fibulin-3 expression levels

There were no significant correlations between survivin and fibulin-3 serum levels within each group (*P>*0.05).Additionally, no significant correlations were found between biochemical markers and tumor stage among the patients with lung cancer and those with MPM (*P>*0.05).

### Diagnostic utility of survivin and fibulin-3 expression levels in patients with respiratory diseases

**Figure 2:**
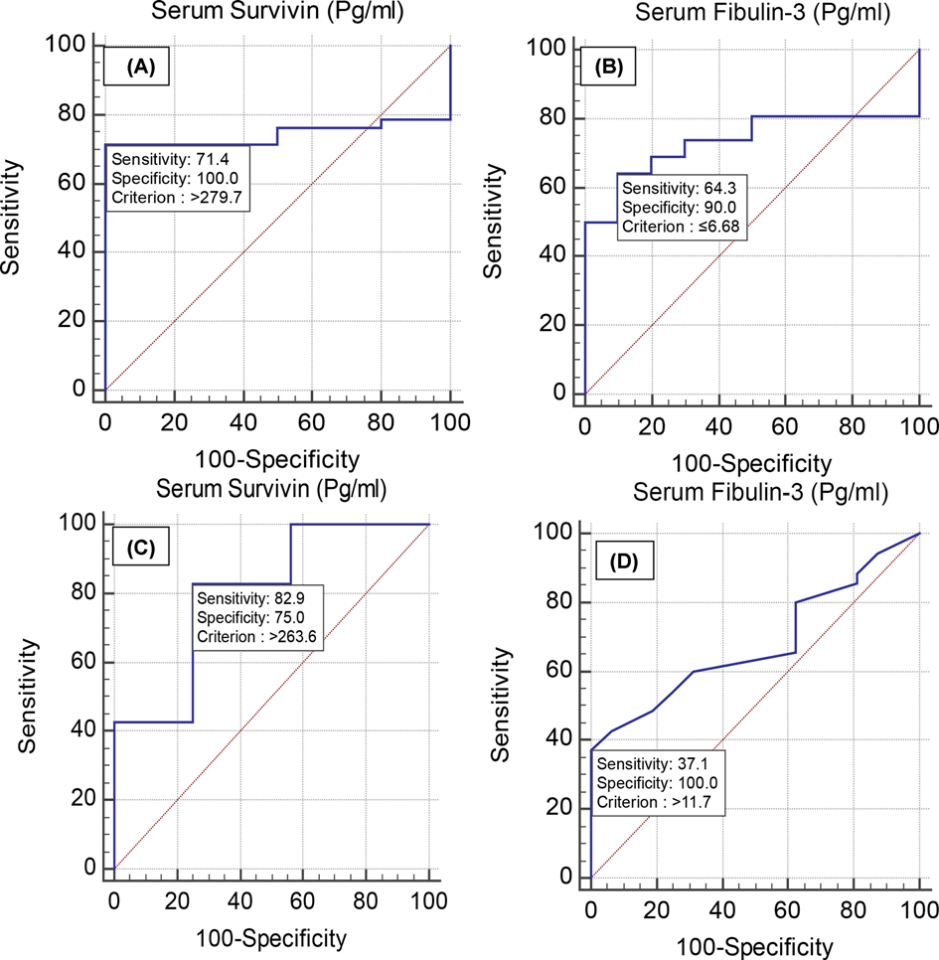
Validity of serum survivin and Serum Fibulin-3 (Pg/mL) in discriminating benign from malignant respiratory diseases Receiver operating characteristic (ROC) curves for serum survivin (**A**) and fibulin-3 (**B**) in predicting lung cancer (*P>*0.05) and (**C** and **D**) in diagnosing malignant pleural mesothelioma (*P>*0.05).

The sensitivity, specificity, PPV, NPV, and AUC for survivin (cut-off point >279.7 pg/ml) in diagnosing lung cancer were 71.43%, 100.0%, 100.0%, 62.5%, and 0.743, respectively; the sensitivity, specificity, PPV, NPV, and AUC for fibulin-3 (cut-off point ≤6.68 pg/ml) were 64.29%, 90.0%, 93.1%, 54.5%, and 0.736, respectively (*P>*0.05) (Figure 2A,B). The sensitivity, specificity, PPV, NPV, and AUC for survivin (cut-off point >263.6 pg/ml) in predicting MPM were 82.86%, 75.0%, 87.9%, 66.7%, and 0.804, respectively, and those for fibulin-3 (cut-off point >11.7 pg/ml) were 37.14%, 100.0%, 100.0%, 42.1%, and 0.680, respectively(*P>*0.05) (Figure 2C,D).

**Figure 3:**
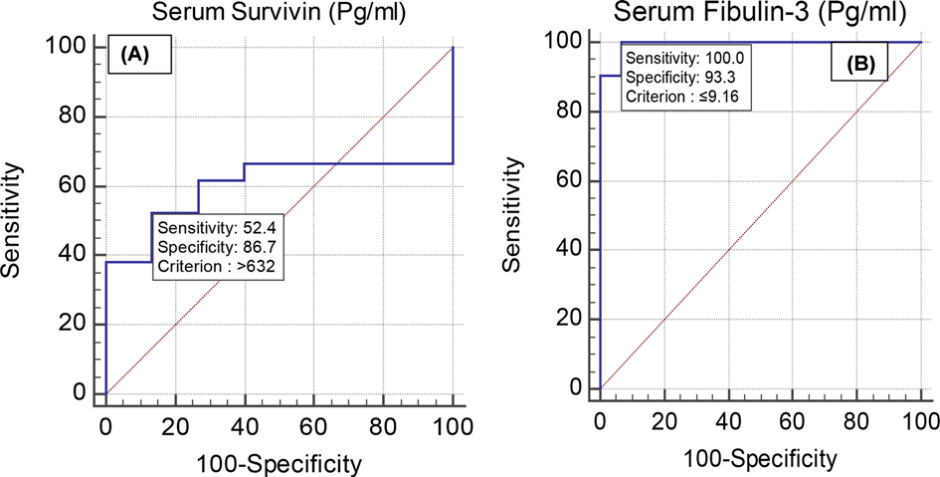
Validity of serum survivin and Serum Fibulin-3 (Pg/mL) in differentiating lung cancer from malignant pleural mesothelioma Receiver Operating Characteristic (ROC) curves for serum survivin (**A**) and fibulin-3 (**B**) in discriminating lung cancer from malignant pleural mesothelioma. Fibulin-3 was superior to survivin (*P<*0.001).

Receiver operating characteristic (ROC) curves for serum survivin and fibulin-3 in discriminating lung cancer from malignant pleural mesothelioma revealed that the sensitivity, specificity, PPV, NPV and AUC for survivin (cut-off point >632 pg/ml) were 52.38%, 86.67%, 84.6%, 56.5%, and 0.603, respectively, while those values for fibulin-3 (cut-off point ≤9.16 pg/ml) were 100.0%, 93.3%, 95.5%, 100.0%, and 0.994, respectively (*P<*0.001) (Figure 3A,B).

## Discussion

Lung cancer is the second leading illness contributing to years of life lost due to premature mortality [19]. The prognosis of MPM is much worse even than other lung cancers. Therefore, the search for additional biomarkers to develop novel strategies for early detection is mandatory.

Although the incidence of adenocarcinoma (50.60%) from 1998 to 2007 was approximately twice that of squamous cell carcinoma (25.24%), the former did not attract as much attention as the latter, which is associated with cigarette smoking according to data from the National Cancer Registry [20]. In the current study, adenocarcinoma was present in 57.1% and squamous cell carcinoma in 42.9% of patients diagnosed with NSCLC. Recently, there has been an increase in the incidence of MPM, classified pathologically into epithelioid (55%), sarcomatoid (15%), or biphasic (30%) types [21,22]. In the current study, 66.7% of MPM patients had epithelioid type mesthothelioma, while the remaining 33.3% had sarcomatoid type MPM.

Survivin over expression in different malignant tumors may indicate a causal association between survivin up-regulation and higher malignant grades or decreased survival rates [23]. In the present study, the expression levels of survivin and fibulin-3 were evaluated in oncologic and non-oncologic diseases of the lung and pleura. Our findings revealed significantly higher circulating and local expression levels of survivin in bronchogenic carcinoma patients compared with in those with benign lung diseases, with non-significant differences in serum levels with regard to the histological types and in correlation with tumor stage. These findings are in agreement with those of previous studies [24–26].

A meta-analysis performed by Duan et al. [27] indicated that survivin expression correlated with tumor stage, but not pathological type or tumor size. They also reported higher survivin expression in patients with NSCLC compared with in normal controls. In addition, Naumnik et al. [28] reported no correlation between serum survivin concentrations and the histological type or staging of lung cancer; in contrast, they reported that survivin concentrations were the same in patients with NSCLC as in healthy individuals. This discrepancy may be attributable to differences in tumor entities and different experimental settings, such as patient selection criteria and study design. In this sense, putative survivin antagonists currently under study show promising antitumoral potential [29].

Notably, our results showed significantly higher serum survivin levels in patients with benign lung diseases than in healthy controls. This confirms similar findings reported by Terasaki et al. [30], who described its high expression as a key mediator of cytoprotection in acute lung injury partly dependent on inhibition of apoptosis.

The findings of the present study revealed significantly lower circulating and local fibulin-3 expression levels in patients with lung cancer than in those with benign lung lesions, with non-significant differences in serum fibulin-3 levels with respect to histological type and no significant correlation to the stage of lung cancer. Additionally, there was a non-significant difference in fibulin-3 serum levels between patients with benign lung lesions and healthy controls. These results were consistent with other studies documenting down-regulation of fibulin-3 in lung cancer due to promoter hypermethylation [10,31,32]. The role of fibulin-3 in cancer is likely to depend on the pathways involved, protein–protein interactions, and tumor microenvironment [33]. In lung cancer, down-regulation of fibulin-3 enhances invasion and metastasis via Wnt/β-catenin activation and matrix metalloproteinase-7 (MMP-7) expression [11].

There were significantly higher circulating and local expression levels of both survivin and fibulin-3 in patients diagnosed with MPM compared with in those who had benign pleural lesions and healthy controls, with no association between survivin and fibulin-3 levels and MPM stage or histological type. Additionally, there were no significant differences in the circulating fibulin-3 between the benign pleural disease group and healthy controls. These findings are supported by many previous studies in the literature [13,34,35,36]. To the best of our knowledge, this is the first study to report the absence of a significant correlation between survivin and fibulin-3 in various benign or malignant respiratory diseases.

The AUC of summary receiver operating characteristics (sROC) is an index that specifies the overall diagnostic value of a test [37]. The existing literature does not address the validity of survivin and fibulin-3 in diagnosing lung cancer and MPM. We reported non-significant differences in the diagnostic validities of survivin and fibulin-3 in diagnosing lung cancer or predicting MPM. Although there are no published data regarding the utility of survivin in diagnosing lung cancer, published humoral fibulin-3 test data revealed high variability among studies, and remains a contentious issue [38]. A subsequent meta-analysis reported that blood fibulin-3 is useful for MPM diagnosis, and its validity was higher than soluble mesothelin-related peptides orosteopontin [38]. Few studies in the literature reported similar findings [37,39].

To the best of our knowledge, this is the first report comparing the validity of serum survivin and fibulin-3 in discriminating lung cancer from MPM. We determined that the sensitivity, specificity, and AUC for survivin were 52.38%, 86.67%, and 0.603, respectively, while those for fibulin-3 were 100.0%, 93.3%, and 0.994, revealing that fibulin-3 is a more valid biomarker in differentiating MPM from lung cancer.

In conclusion, the present study showed that serum survivin and fibulin-3 could be used as effective diagnostic markers for lung cancer and MPM. No significant differences in the diagnostic validity of serum survivin and fibulin-3 were found in predicting lung cancer or MPM from corresponding benign lesions, although serum fibulin-3 was more useful in differentiating lung cancer from MPM. Furthermore, the present study confirms the lack of association between survivin and fibulin-3 expression levels with histological type and tumor stage.

## Study Limitations

The main limitation of this study was the relatively small sample size recruited from a single hospital center.

## Trial registration

ClinicalTrials.gov Identifier: NCT04413292: https://clinicaltrials.gov/ct2/show/NCT04413292, retrospectively registered.

## Supplementary Material

Supplementary Figures S1-S2

## Data Availability

The data sets used and analyzed in this study are available upon reasonable request.

## Abbreviations

BCIP/NBT: 5-bromo-4-chloro-3-indolyl phosphate/nitro blue tetrazolium
CBC: complete blood count
EMT: epithelial–mesenchymal transition
ESR: erythrocyte sedimentation rate
GSK3β: glycogen synthase kinase 3β
IAP: inhibitors of the apoptosis protein
MMP-7: matrix metalloproteinase-7
MPM: malignant pleural mesothelioma
NSCLC: non-small cell lung carcinoma
PVDF: polyvinylidene difluoride membrane
RIPA buffer: radioimmunoprecipitation assay buffer
TBS: Tris-buffered saline
TBST: Tris-buffered saline with tween
TGF-β: transforming growth factor beta
