# Supplementary material for: Circulating and local nuclear expression of survivin and fibulin-3 genes in discriminating benign from malignant respiratory diseases: correlation analysis
Source: Biosci Rep. 2021 Jan 6;41(1):BSR20203097. doi: 10.1042/BSR20203097 (PMC7789905; doi:10.1042/BSR20203097)
Supplement: Supplementary Figures S1-S2 [file BSR-2020-3097_supp.pdf]

Supplemntary materials

Circulating and local nuclear expression of survivin and fibulin-3 genes in discriminating benign from malignant respiratory diseases: Correlation analysis

Running title: Survivin and fibulin-3 in respiratory diseases

Mohammed H. Hassan[1,*] Sawsan Abuhamdah [2,3] Mohamed Abdel-Bary [4], Mohammed Wahman[5], Tarek Hamdy Abd-Elhamid[6], Morris Beshay[7], Karam Mosallam[4], Bakheet E.M. Elsadek[8]

[1]Department of Medical Biochemistry, Faculty of Medicine, South Valley University, Qena, Egypt.

[2]College of Pharmacy, Al Ain University, Abu Dhabi, UAE.

[3]Department of Biopharmaceutics and Clinical Pharmacy, Faculty of Pharmacy, The University of Jordan, Amman, Jordan.

[4]Department of Cardio-Thoracic Surgery, Faculty of Medicine, South Valley University, Qena, Egypt.

[5]Department of Oncology and Nuclear Medicine, Faculty of Medicine, South Valley University, Qena, Egypt.

[6]Department of Histology and Cell Biology, Faculty of Medicine, Assiut University, Assiut, Egypt.

[7]Department of General Thoracic Surgery, Protestant Hospital of Bethel Foundation, Burgsteig 13, Bielefeld, Germany.

[8]Department of Biochemistry, Faculty of Pharmacy, Al-Azhar University, Assiut Branch, 71524 Assiut, Egypt.

Corresponding author: *To whom correspondence should be addressed:

Dr. Mohammed H. Hassan, Associate Professor of Medical Biochemistry, Faculty of Medicine, South Valley University, Qena, Egypt.

E-mail: Mohammedhosnyhassaan@yahoo.com;

mohammedhosnyhassaan@med.svu.edu.eg; Tel.: +201098473605

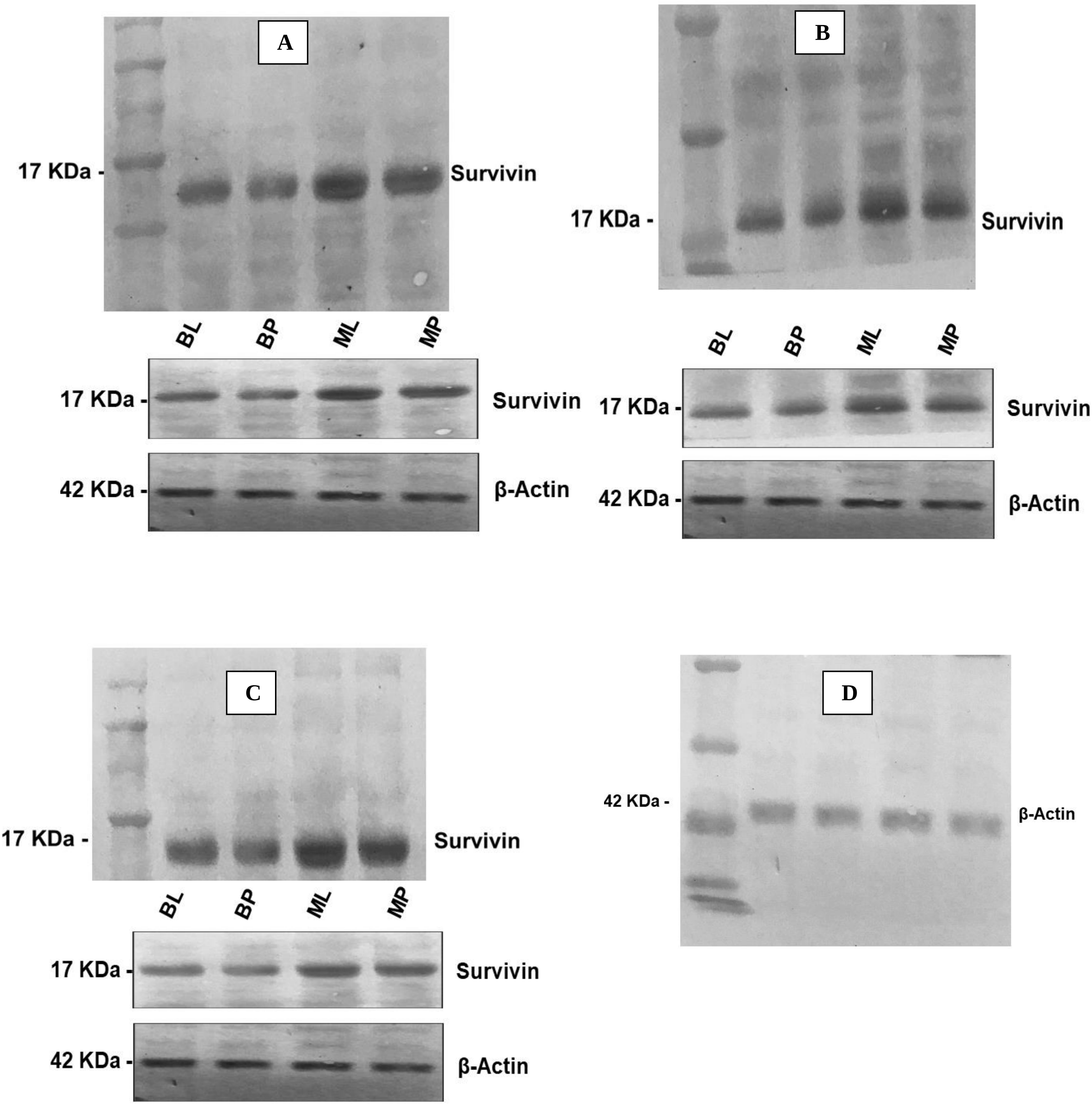

Supplementary Figure1. Full uncropped and unedited versions of the western blots for survivin expression (A): First run; (B) second run; (C): third run; (D) β-actin; from patients with various benign and malignant respiratory diseases to assure the reproducibility of results. BL, benign lung diseases; BP, benign pleural diseases; ML, malignant lung diseases; MP, malignant pleural diseases.

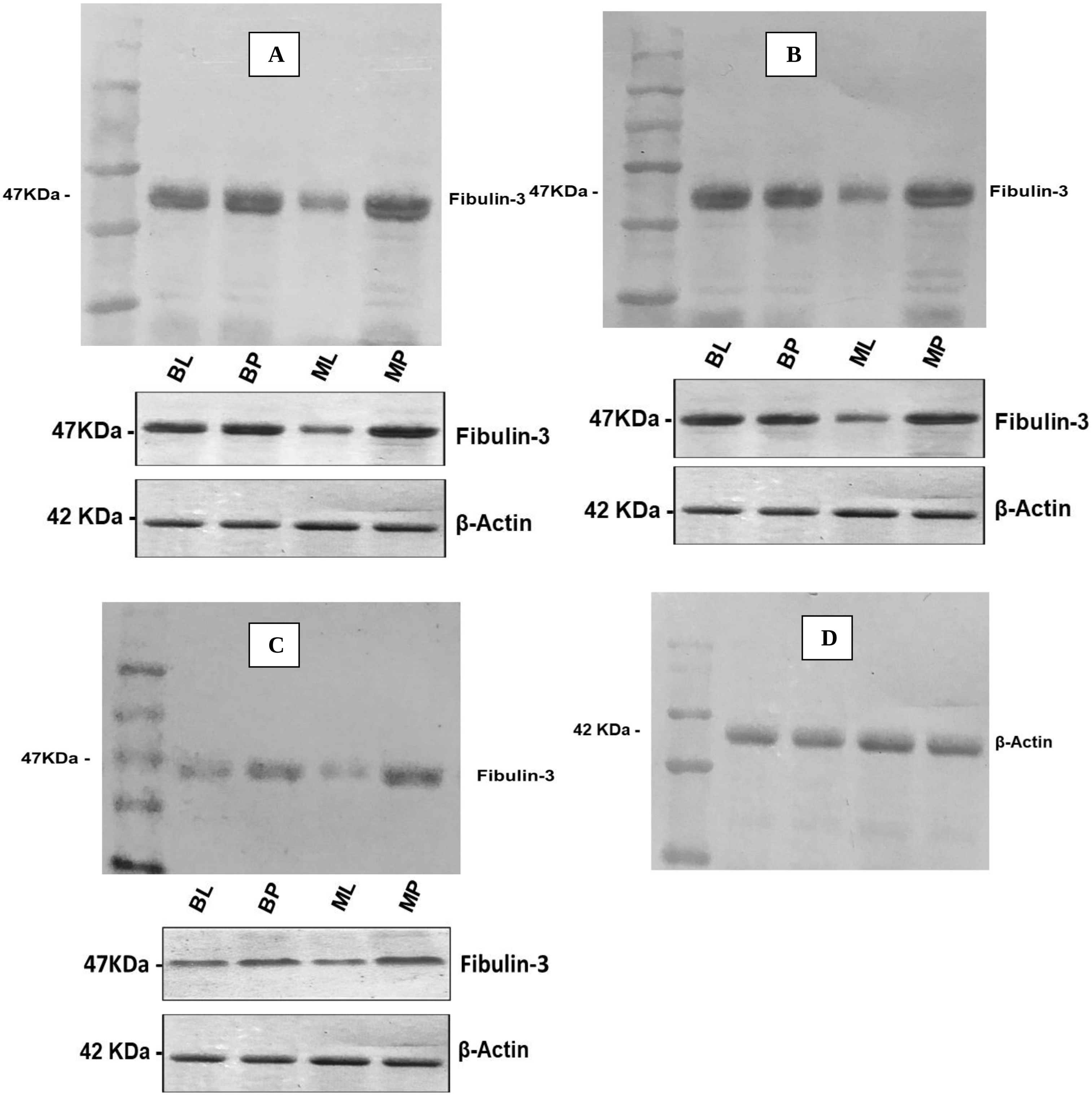

Supplementary Figure 2. Full uncropped and unedited versions of the western blots for fibulin-3 expression (A): First run; (B) second run; (C): third run; (D) β-actin; from patients with various benign and malignant respiratory diseases to assure the reproducibility of results. BL, benign lung diseases; BP, benign pleural diseases; ML, malignant lung diseases; MP, malignant pleural diseases.
